# The pepper virome: natural co-infection of diverse viruses and their quasispecies

**DOI:** 10.1186/s12864-017-3838-8

**Published:** 2017-06-08

**Authors:** Yeonhwa Jo, Hoseong Choi, Sang-Min Kim, Sun-Lim Kim, Bong Choon Lee, Won Kyong Cho

**Affiliations:** 1 Department of Agricultural Biotechnology, College of Agriculture and Life Sciences, Seoul National University, Seoul, 151-921 Republic of Korea; 2 Crop Foundation Division, National Institute of Crop Science, RDA, Wanju, 55365 South Korea; 3 The Taejin Genome Institute, Gadam-gil 61, Hoeongseong, 25239 Republic of Korea

**Keywords:** Cultivar, Pepper, Plant virus, Quasispecies, Virome

## Abstract

**Background:**

The co-infection of diverse viruses in a host plant is common; however, little is known about viral populations and their quasispecies in the host.

**Results:**

Here, we report the first pepper viromes that were co-infected by different types of viral genomes. The pepper viromes are dominated by geminivirus DNA-A followed by a novel carlavirus referred to as Pepper virus A. The two pepper cultivars share similar viral populations and replications. However, the quasispecies for double-stranded RNA virus and two satellite DNAs were heterogeneous and homogenous in susceptible and resistant cultivars, respectively, indicating the quasispecies of an individual virus depends on the host.

**Conclusions:**

Taken together, we provide the first evidence that the host plant resistant to viruses has an unrevealed antiviral system, affecting viral quasispecies, not replication.

**Electronic supplementary material:**

The online version of this article (doi:10.1186/s12864-017-3838-8) contains supplementary material, which is available to authorized users.

## Background

Pepper plants in the *Capsicum* species, which are members of the Solanaceae family, have been cultivated worldwide and consumed as fresh fruits or powdered spices [1]. A wide range of pathogens, including viruses, cause serious diseases in pepper plants. In particular, cultivated pepper plants are susceptible to diverse plant viruses, including DNA and RNA viruses [2]. Out of 68 known virus species that infect peppers, about 20 cause viral diseases in pepper plants [2]. Among the known RNA viruses that infect pepper plants, viruses in the genera *Potyvirus*, *Cucumovirus*, *Polerovirus*, and *Crinivirus* have been reported [2]. Of the known DNA viruses that infect pepper plants, members of the genus *Begomovirus*, in the family *Geminiviridae*, are frequently reported. Begomoviruses transmitted by *Bemisia tabaci* are very destructive and emerging viruses in the production of pepper [3].

Next-generation sequencing (NGS) enables us to identify known as well as novel viruses in various plant species [4]. In the case of pepper plants, several known viruses, including *Pepper vein yellows virus* and *Bell pepper endornavirus* (BPEV), and a novel virus, *Pepper yellow leaf curl virus*, have been identified by Illumina NGS techniques [5–8].

A wide range of viruses display a significant level of genetic diversity within the host by replicating with high mutation rates [9]. Viral populations are often composed of mutant clouds rather than a uniform viral genome with the same nucleotide sequence, which are referred to as viral quasispecies [10]. It is known that high mutation rates as well as genetic recombination or reassortment are important factors for viral quasispecies [10, 11]. Studies on the quasispecies of medically important RNA viruses have been intensively conducted [9]. In the case of plant RNA viruses, a previous study using three different viruses demonstrated that the quasispecies of many RNA viruses are influenced by host-virus interactions [12]. In addition, some plant DNA viruses belonging to begomoviruses, such as *Tomato yellow leaf curl virus* (TYLCV), also exhibit high mutation rates, suggesting quasispecies of single-stranded (ss) DNA viruses in the host [13].

Several previous studies have reported that many plants are often co-infected by different plant viruses and viroids [14–16]. Plants, including fruit trees, ornamental plants, bulb plants, and tubers, are usually cultivated by vegetative reproduction, such as clonal propagation and grafting. Therefore, rates of virus co-infection are much higher than in plants propagated by seeds. For instance, previous studies have reported the co-infection of eight viruses and two viroids in a single grapevine cultivar [14], nine different RNA viruses in garlic bulbs [17], and 11 RNA viruses in a single sweet potato cultivar [18].

Recently, we screened available pepper transcriptomes to identify BPEV-associated sequences [19]. Out of 77 screened pepper transcriptomes, two pepper transcriptomes were co-infected by different DNA and RNA viruses. In this study, we carried out systematic bioinformatics analyses to identify viruses, including a novel RNA virus that infects pepper plants. In addition, we revealed viral populations and quasispecies in two different pepper cultivars.

## Results

### De novo transcriptome assembly and virus identification

**Fig. 1:**
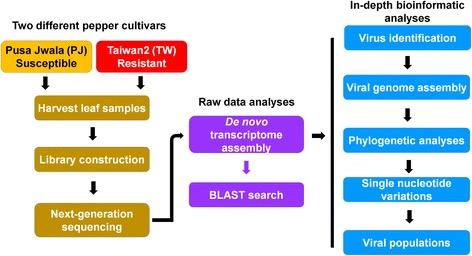
Experimental scheme for the study of two pepper viromes. Transcriptomes from two different cultivars – Pusa Jwala (PJ) and Taiwan2 (TW) – were analyzed for the study of pepper viromes. Detailed data analyses are described in Methods

**Fig. 2:**
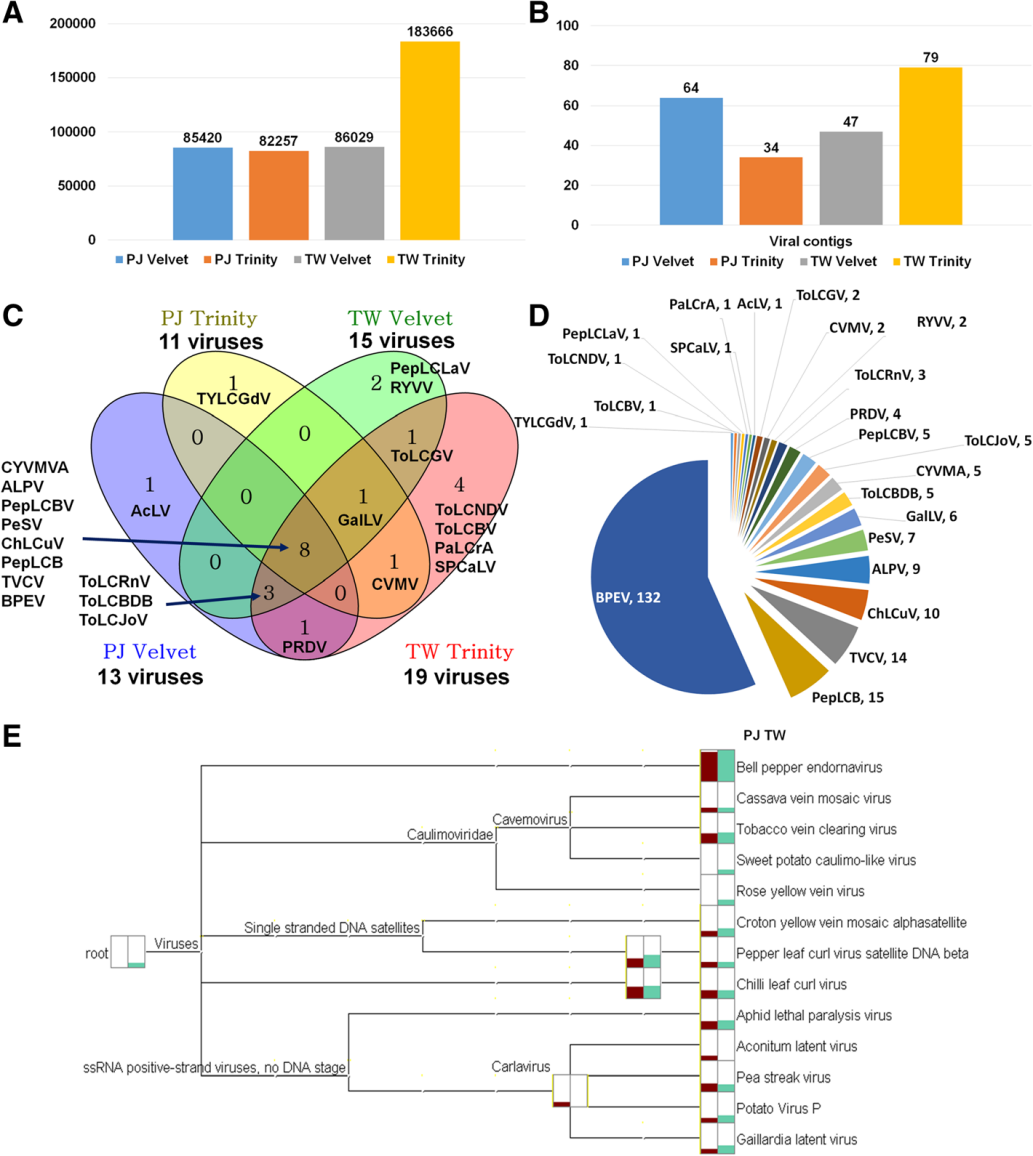
Identification of viruses infecting two pepper cultivars using pepper transcriptome data. **a** Numbers of contigs in four different datasets that were de novo-assembled from two different libraries named PJ and TW by two different assemblers: Velvet and Trinity. **b** Numbers of virus-associated contigs in four different datasets. **c** Venn diagram displays the numbers of identified viruses in four different datasets. **d** Pie chart displays numbers of contigs assigned to respective virus identified from four different datasets. **e** Classification of identified viruses based on taxonomy using MEGAN program. *Dark red* and *green* colored bars indicate the amount of contigs associated with the assigned virus

The two different transcriptomes were derived from Pusa Jwala (PJ) and Taiwan2 (TW) cultivars, which were susceptible and resistant to various viruses, respectively [20, 21]. Detailed experimental scheme can be found in Fig. 1. The raw data size of TW (7.3 Gb) was about two times that of PJ (3.8 Gb) (Additional file 1: Table S1). Initially, the raw data were de novo assembled by Velvet programs. We again de novo assembled two pepper transcriptomes using Trinity. The number of assembled contigs ranged from 82,257 (PJ by Trinity) to 183,666 (TW by Trinity) (Fig. 2a). Each dataset was subjected to a BLAST search against the viral reference database. We identified several virus-associated contigs that ranged from 34 to 79 contigs (Fig. 2b) (Additional file 1: Tables S2 and S3). We compared the number of identified virus-associated contigs from four different datasets (Fig. 2c). TW by Trinity identified 19 viruses followed by TW by Velvet (15 viruses), PJ by Velvet (13 viruses), and PJ by Trinity (11 viruses). Eight viruses – *Croton yellow vein mosaic virus* alphasatellite (CYVMVA), *Aphid lethal paralysis virus* (ALPV), *Pepper leaf curl Bangladesh virus* (PepLCBV), *Pea streak virus* (PeSV), *Chilli leaf curl virus* (ChiLCV), *Pepper leaf curl virus* betasatellite (PepLCVB), *Tobacco vein clearing virus* (TVCV), and *Bell pepper endornavirus* (BPEV) – were commonly identified in four datasets. Several viruses were specifically identified in each dataset. We combined all virus-associated contigs from four different datasets (Fig. 2d). BPEV-associated contigs (132 contigs) were the most abundant, followed by PepLCVB (15 contigs), TVCV (14 contigs), and ChiLCV (10 contigs). We classified the identified virus-associated contigs according to virus taxonomy, resulting in 13 different virus species (Fig. 2e). They can be further divided into four different types based on their genome types (Additional file 1: Table S4). For example, BPEV is a double-stranded (ds) RNA virus, and four viruses, including TVCV, are dsDNA viruses. In addition, we identified several ssDNA viruses belonging to the family *Geminiviridae* (ChiLCV DNA-A, PepLCV betasatellite, and CYVMV alphasatellite). Of five ssRNA viruses, ALPV is an aphid-infecting virus (ALPV) in the family *Dicistroviridae*, and the other four viruses – *Aconitum latent virus* (AcLV), PeSV, *Potato virus P* (PVP), and *Gaillardia latent virus* (GalLV) – are members of the genus *Carlavirus*.

### De novo assembly of viral genomes

**Fig. 3:**
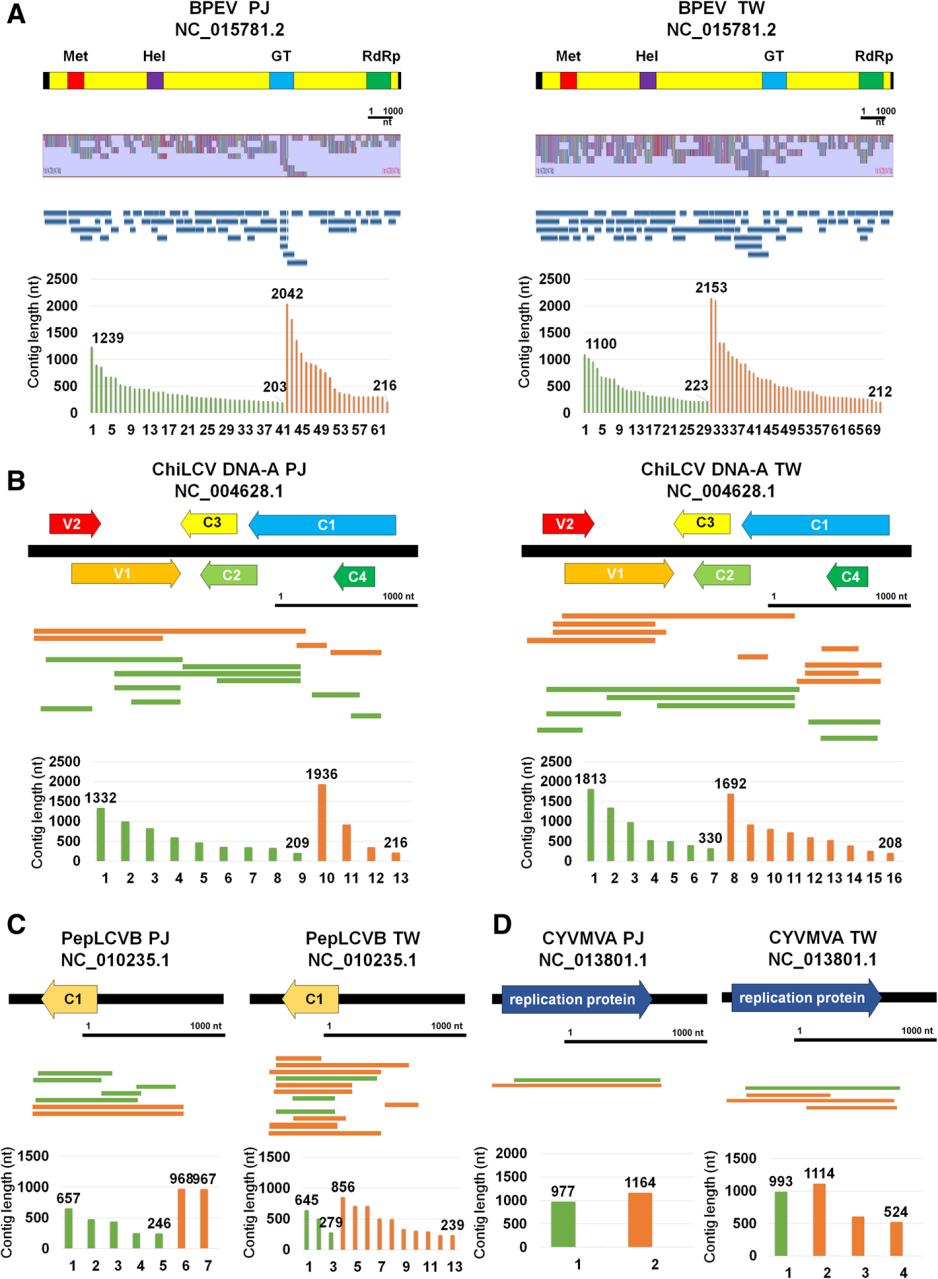
Assembly of a consensus viral genome sequence for four identified viruses. Assembly of consensus viral genome sequences for BPEV (**a**), ChiLCV DNA-A (**b**), PepLCVB (**c**), and CyVMVA (**d**). Contigs associated with corresponding virus were aligned using BWA and visualized by Tablet program. *Black bar* represents 1000 nucleotides. *Green* and *orange* colored *lines* and *bars* indicate contigs assembled by Velvet and Trinity, respectively. The numbers in the *bar* charts indicate lengths for the longest and shortest contigs, respectively. The genome organizations for four identified viruses were manually drawn based on the reference viral genome annotation. In case of BPEV, conserved domains were indicated. For circular DNA viruses, linear genome types were drawn with corresponding genes

To assemble viral genomes using transcriptome data, we mapped the identified virus-associated contigs on the respective reference virus genome. Of the identified viruses, BPEV-associated contigs were enriched. The sizes of BPEV-associated contigs ranged from 2153 nt to 203 nt (Fig. 3a). In general, the sizes of the contigs assembled by Trinity were longer than those assembled by Velvet. For example, the longest contig by Velvet was 1239 nt, while the longest contig by Trinity was 2042 nt in PJ. However, more contigs were assembled by Velvet (41 contigs) than Trinity (20 contigs). A total of 61 and 69 BPEV-associated contigs from PJ and TW, respectively, were mapped on the reference genome and covered most regions of the BPEV genome with several unmapped regions (Fig. 3a). In addition, seven contigs from PJ and TW, respectively, were aligned on the TVCV reference genome (Additional file 2: Figure S1). Many contigs associated with TVCV were mapped on the polyprotein region of TVCV.

We identified several contigs associated with DNA virus components. A total of 13 and 16 contigs from PJ and TW, respectively, were closely associated with ChiLCV DNA-A (Fig. 3b). The lengths of ChiLCV DNA-A-associated contigs ranged from 1936 nt to 208 nt. The contigs were aligned on the complete sequences of five genes for ChiLCV DNA-A except the C1 gene. In addition, we identified seven and 13 contigs associated with PepLCVB from PJ and TW, respectively (Fig. 3c). The lengths of the contigs associated with PepLCVB ranged from 968 nt to 239 nt. Moreover, six CYVMVA-associated contigs, which fully cover a gene-encoding replication protein, were identified (Fig. 3c). The size of CYVMVA-associated contigs ranged from 1164 nt to 524 nt. Using contigs aligned to the reference genome, we obtained partial consensus sequences for DNA virus components. We aligned the obtained nucleotide and protein sequences for the PepLCVB C1 gene and CYVMVA replication gene with the respective reference sequence (Additional file 2: Figures S2 and S3). The sequence alignment showed the newly identified PepLCVB and CYVMVA might be novel sequences displaying many polymorphisms with the reference sequences.

### Identification and complete genome assembly of a novel ssRNA virus

**Fig. 4:**
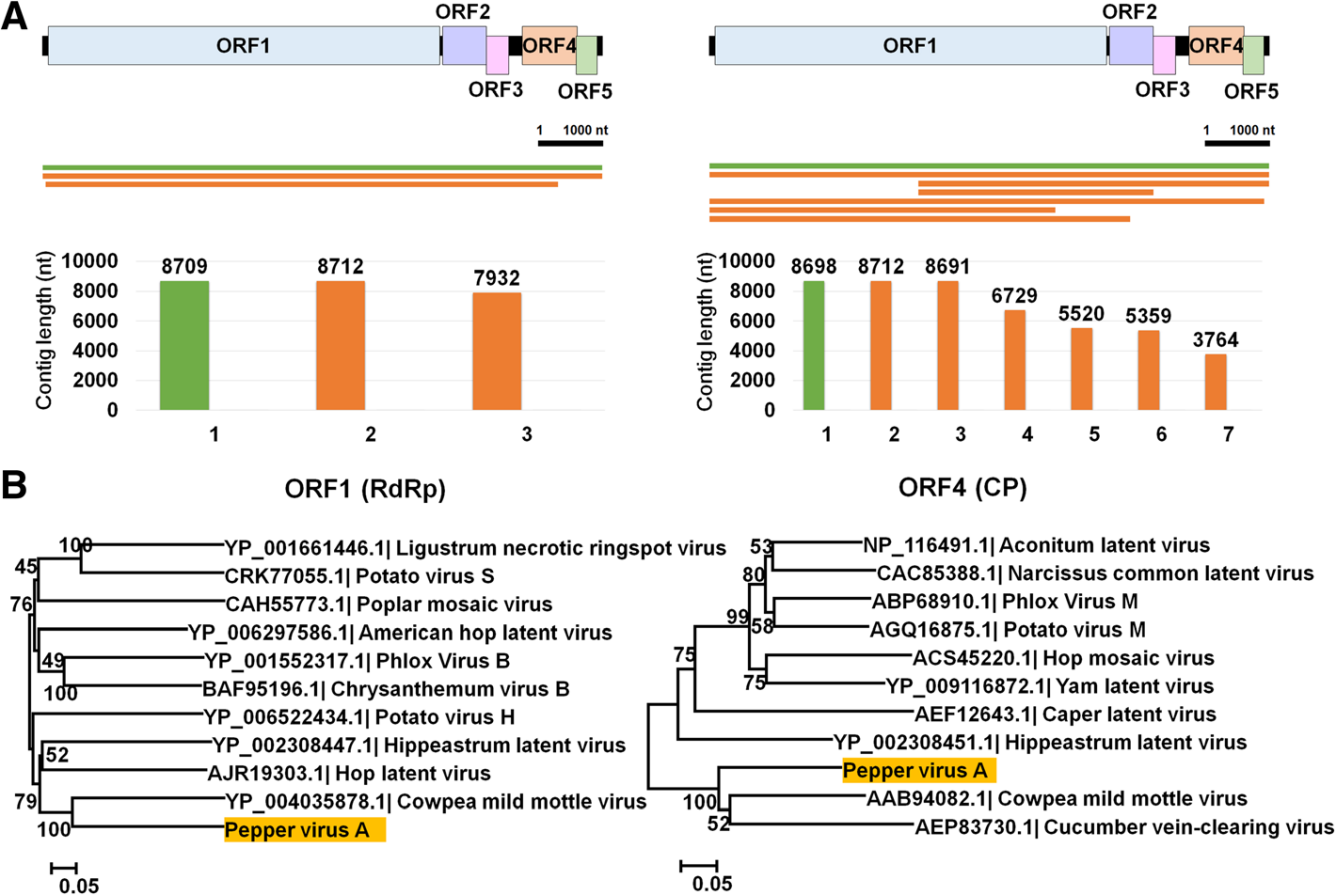
Identification of a novel virus referred to as PepVA. **a** Genome organization of newly identified PepVA, contigs associated with PepVA were aligned on the PepVA genome, and lengths of contigs associated with PepVA.* Green* and *orange* colored lines and bars indicate contigs assembled by Velvet and Trinity. **b** Phylogenetic relationships of ORF1 and ORF4 with respective other homologous viral proteins for PepVA

BLASTX search and conserved domain prediction revealed that the ten contigs associated with ssRNA viruses, including AcLV, PeSV, PVP, and GalLV, were sequences derived from a novel virus. The five assembled contigs were nearly complete or complete genome sequences for a novel ssRNA virus (Fig. 4a). The novel virus encodes five open reading frames (ORFs) (Fig. 4a). ORF1 encodes a putative RNA-dependent RNA polymerase. ORF2 and ORF3 encode proteins similar to triple-gene-block (TGB) proteins named TGB1 and TGB2. ORF4 encodes a coat protein, while ORF5 encodes a putative nucleic acid-binding protein. We named the newly identified virus Pepper virus A (PepVA) (Accession No. KU726694). A BLAST search using individual ORF sequences revealed that PepVA belongs to the genus *Carlavirus* in the family *Betaflexiviridae*. Phylogenetic analyses revealed that PepVA is closely related to *Cowpea mild mottle virus*, followed by *Cucumber vein-clearing virus* and *Hippeastrum latent virus* (Fig. 3b and Additional file 2: Figure S4).

### Phylogenetic relationships of identified viruses

**Fig. 5:**
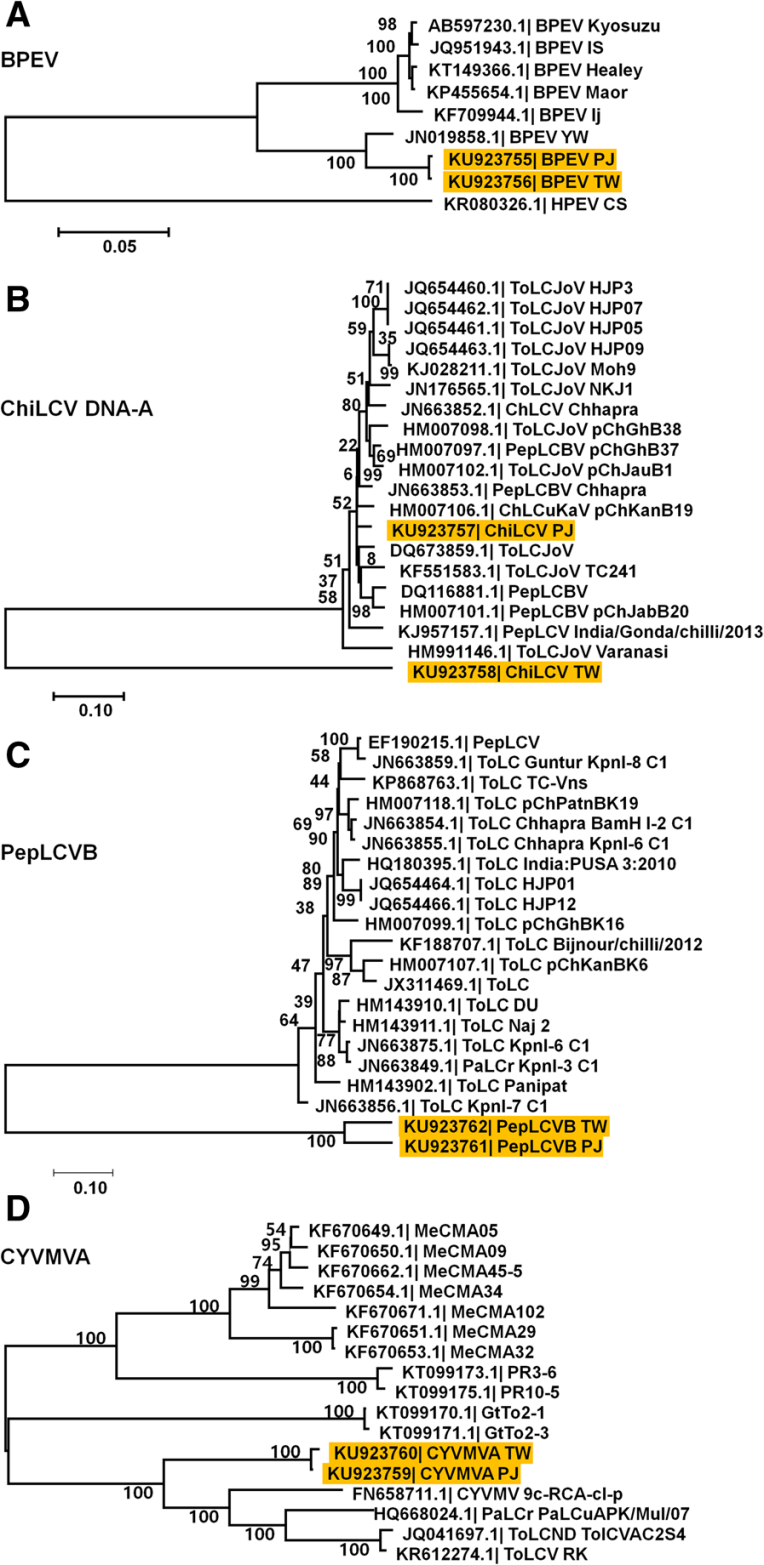
Phylogenetic relationships of four identified viruses. Phylogenetic trees for BPEV (**a**), ChiLCV DNA-A (**b**), PepLCVB (**c**), and CYVMVA (**d**) were constructed by MEGA6 program. Consensus genome sequences obtained from PJ and TW cultivars as well as respective known genome sequences for individual virus were used for phylogenetic tree construction

To reveal the phylogenetic origin of the identified viruses, we generated phylogenetic trees for each virus using the assembled nearly complete viral genomes or partial sequences along the respective homologous viral genome or sequence. A representative single consensus sequence for PJ and TW has been used for the phylogenetic analysis of each virus. Although the BPEV isolates PJ and TW were identified from pepper plants, two isolates were closely related to the BPEV isolate YW identified from bell pepper plants (Fig. 5a). In the case of ChiLCV DNA-A, the isolate PJ was closely related to other isolates from ChLCuV, PepLCV, and ToLCV, while the isolate TW was distantly related to the other DNA-A sequences of geminiviruses, indicating a possible new geminiviral DNA-A sequence (Fig. 5b). In the case of PepLCVB and CYVMVA, both isolates from PJ and TW were closely related (Fig. 5c and d). However, they are somehow distantly related to the satellite sequences of other geminiviruses. Therefore, the alphasatellite and betasatellite sequences from PJ and TW might be novel satellite DNA sequences based on sequence alignment and phylogenetic analyses.

### Single nucleotide variations of identified viruses in two different pepper cultivars

**Fig. 6:**
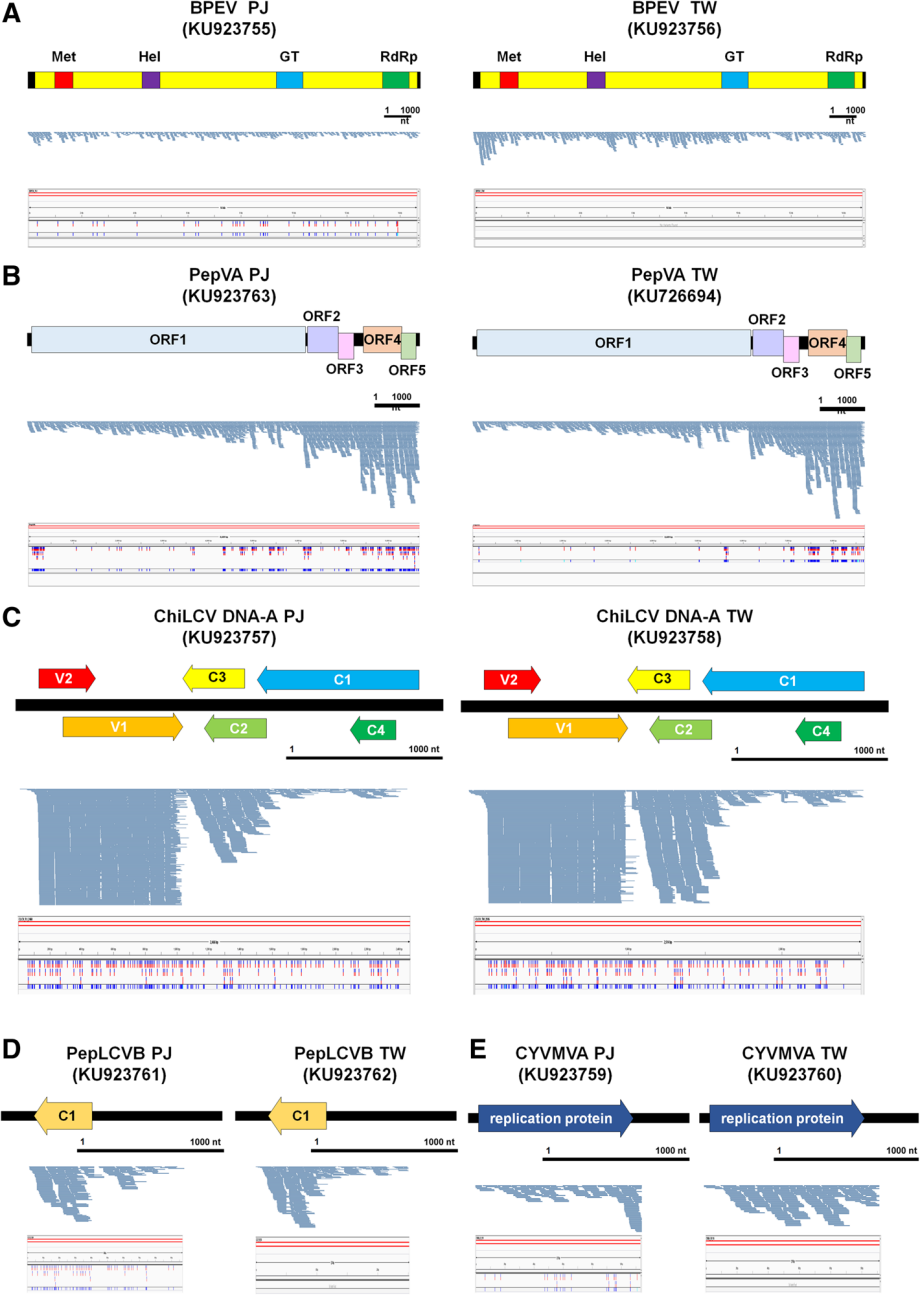
Analysis of single nucleotide variations for five identified viruses. Single nucleotide variations for BPEV (**a**), PepVA (**b**), ChiLCV DNA-A (**c**), PepLCVB (**d**), and CYVMVA (**e**) were identified by SAMtools and visualized by Tablet program. Genome organization for each virus was drawn based on respective reference sequence. Raw sequence data were mapped on the respective consensus viral genome sequences from PJ and TW, respectively. *Red* and *blue bars* indicate positions of identified single nucleotide variations on the viral genome

To examine the quasispecies of an individual virus, we analyzed single nucleotide variations (SNVs) for each virus using assembled consensus genome sequences in an individual cultivar (Additional file 1: Tables S5–S14). For BPEV, 40 SNVs were identified in the isolate PJ, while no SNVs were detected in the isolate TW (Fig. 6a). For PepVA, 269 SNVs were detected in the isolate PJ and 130 SNVs were identified in the isolate TW (Fig. 6b). Many SNVs were identified in the C-terminal region of PepVA, which correlates with the number of reads mapped on the PepVA genome. Although the genome size of ChiLCV DNA-A is relatively smaller than that of other ssRNA and dsRNA viruses, the number of identified SNVs was very high. For example, 204 and 197 SNVs were identified for PJ and TW, respectively (Fig. 6c). The SNVs were evenly distributed along the DNA-A sequences. For PepLCVB, 70 SNVs were identified in the PJ, while no SNVs were identified in the TW (Fig. 6d). Again, 23 SNVs were identified in the PJ and no SNVs were identified in the TW for CYVMVA (Fig. 6e).

**Fig. 7:**
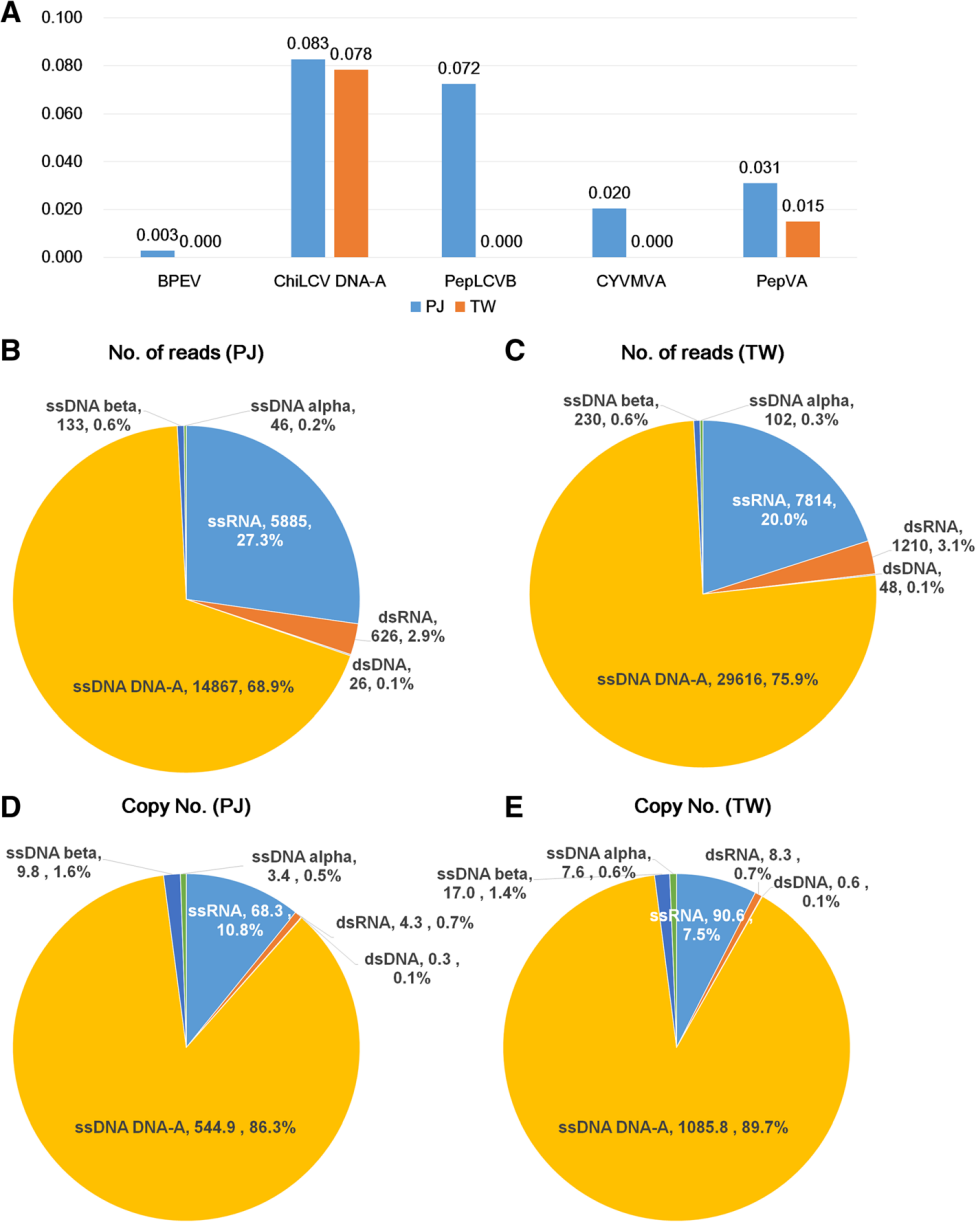
Mutation rates of five viral genomes and viral populations. **a** Mutation rates for five viral genomes in PJ and TW cultivars. Numbers of sequence reads associated with respective identified viruses in PJ (**b**) and TW (**c**). Virus copy number for each identified virus in PJ (**d**) and TW (**e**)

Next, we examined the mutation rates for each virus in an individual cultivar (Fig. 7a). Of the five examined viral genomes, ChiLCV DNA-A displays the highest mutation rates (e.g. 0.083 and 0.078 for PJ and TW, respectively), followed by PepLCVB (0.072) in PJ. The mutation rate for PepVA in PJ (0.031) was twice that in TW (0.015). BPEV exhibited the lowest mutation rates (e.g. 0.003 and 0 for PJ and TW, respectively). Surprisingly, we found that the BPEV, PepLCVB, and CYVMVA isolates in the TW cultivar did not show SNVs, indicating that only homogenous sequences for the three viral genomes in the resistant cultivar were present.

### Viral population and the amount of virus replication

Our results showed that at least three different types of plant viruses were co-infected in the PJ and TW cultivars. We examined the amount of viral RNAs and copy numbers of the individual viral genome in each cultivar (Additional file 1: Table S15). The amount of viral reads associated with DNA-A (68.9% and 75.9% in PJ and TW, respectively) was dominant, followed by ssRNA (27.3% and 20% in PJ and TW, respectively) and dsRNA (2.9% and 3.1% in PJ and TW, respectively) (Fig. 7b and c). The number of reads associated with alphasatellite and betasatellite DNAs ranging 0.1% to 0.6% were very low. Based on the copy number of each viral genome, the portion of ssDNA was increased to 89.3% and 89.7% in PJ and TW, respectively, while the portion of dsRNA with the largest genome among the identified viruses was decreased to 0.7% in both cultivars (Fig. 7d and e). The portion of ssRNA was decreased, while the portion of satellite DNAs was increased.

## Discussion

In this study, we conducted systemic bioinformatics analyses to reveal viral populations and their quasispecies in two different pepper plants, which are susceptible and resistant to various viruses, respectively. We will discuss how our in silico analyses using transcriptome data were very effective for discovering a wide range of new biologically significant results.

Although pepper plants are susceptible to a large number of RNA and DNA viruses, the coinfection of diverse DNA and RNA viruses in pepper plants was surprising. In addition, it was very difficult to reveal individual viral genomes from complex viral populations showing a high level of genetic diversity. For this, much experience and many techniques are required. An important factor for identifying viruses using NGS data is the length of viral contig. For example, BLAST results using short lengths of contigs revealed various viruses that are members in the same genus, although these contigs were derived from a single virus genome. For example, all contigs associated with AcLV, PeSV, PVP, and GalLV identified by BLAST search in our study were finally revealed as sequences derived from a novel virus, PepVA. Therefore, the BLAST results from partial sequences sometimes lead to the wrong interpretation for virus identification. Therefore, we recommend complete or nearly complete viral genome sequences for virus identification using BLAST search.

The two libraries in this study were constructed based on oligo(dT) primers to amplify the mRNAs of pepper. Thus, the identification of ssRNA with poly(A) tail, such as PepVA, was not surprising. However, the identification of RNA viruses without poly(A) tail, such as BPEV, as well as the identification of DNA viruses, such as TVCV, and several geminiviral DNA-A, alphasatellite, and betasatellite sequences from the plant transcriptomes is the first reported to our knowledge. Recently, several studies have shown that RNA viruses without poly(A) tail can be recovered by RNA-Seq data [14, 19]. Previous studies have adapted the enrichment of circular DNAs by PCR followed by NGS to identify DNA viruses and their satellite DNAs [22, 23]. In addition, plant RNA and DNA viruses were de novo assembled by small RNA sequencing [24]. Our data showed that geminivirus segments, such as DNA-A and satellite DNAs, can also be identified from mRNA transcriptome data, which can fully cover most regions of virus-encoded genes. Based on our results, we suggest the possible application of mRNA transcriptome data for plant virus-associated studies.

We identified ten contigs with no close relatives to known viruses. However, conserved domain prediction and phylogenetic studies have identified a novel virus tentatively named PepVA belonging to the genus *Carlavirus*. Due to the poly(A) tail of PepVA, it was possible to assemble two complete genomes of PepVA from PJ and TW using only transcriptome data.

Recent studies have demonstrated that plant transcriptome data can be usefully applied in the assembly of complete or nearly complete genomes for several viruses and viroids [6, 14, 25]. In our experience, several factors, such as viral sequence coverage, the amount of viral replication, and sequencing methods, are important for the assembly of viral genomes. The number of sequence reads from paired-end sequencing is twice that from single-end sequencing. In general, paired-end sequencing is optimal for the de novo assembly of plant transcriptomes as well as viral genomes. In this study, we used two well-known de novo assemblers – Trinity and Velvet – for de novo transcriptome assembly and compared their viral genome assembly ability. As we have demonstrated in our recent study [26], Trinity was far superior for de novo assembly of the virus genome with longer contigs compared with Velvet. In general, velvet generated several short-length contigs, which is not optimal for virus genome assembly; however, the contigs generated by Velvet can be useful for the identification of viruses with low titers. Based on our results, the optimal combination for virus identification and viral genome assembly from plant transcriptomes might be de novo assembly using Trinity with paired-end sequencing. However, we also highly recommend assembling transcriptomes with two methods for virus-associated studies. Previously, many studies have used conventional PCR followed by Sanger sequencing to examine quasispecies of plant RNA and DNA viruses in different hosts [12, 26, 27]. Although the sizes of viral genomes are relatively small, several fragments for a target viral genome should be amplified instead of amplifying a complete viral genome. Furthermore, to understand the comprehensive nature of quasispecies for target viruses, a high level of sequence coverage is necessary. The sequence coverage through the conventional methods composed of PCR and Sanger sequencing is relatively low, while several NGS-based approaches produce numerous sequence reads, which are enough to reveal the quasispecies of target viruses. Therefore, several studies associated with viral quasispecies including viroids have used diverse NGS techniques instead of the PCR-based conventional methods [28–31].

The same list of diverse viruses has been infected in two different pepper transcriptomes, indicating that the two cultivars were grown in the same field. The presence of ALPV suggests the sampled pepper plants were severely damaged by aphids, which are vectors for many plant viruses [32]. In addition, the identification of geminiviral components in pepper plants suggests the transmission of geminivirus by *Bemisia tabaci* [33]. The co-infection of diverse viruses in pepper plants indicates that these plants were naturally grown without any disease control by humans. Therefore, these samples are good examples for studying the natural co-infection of diverse viruses in different pepper cultivars and their quasispecies. A previous study demonstrated that BPEV and TVCV are transmitted by seeds [34, 35]. In addition, a recent study indicates the possible transmission of a geminivirus into tomatoes by seeds [36]. Based on previous results, the co-infection of diverse viruses in pepper plants might also be mediated by not only insect vectors, but also seeds.

In general, the co-infection of various viruses in the host might be harmful for the host. For example, a previous study showed that the mixed infection of two geminiviruses in the pepper plants exhibited a synergistic interaction with increased disease symptoms [37]. Although viruses are regarded as pathogens, some viruses might be good, which means that they are symbiotic partners in the host and play beneficial roles in the plant [38, 39]. In contrast, it is highly possible that some co-infected viruses do not have any association with the host disease symptoms.

The two different pepper cultivars, PJ and TW, have been demonstrated to be susceptible and resistant to diverse viruses, including *Pepper leaf curl virus* (PepLCV) infection in field conditions [20, 21]. Unexpectedly, we found a significant difference in mutation rates, but not in viral replication. The most surprising result was that out of the five examined viral genomes, BPEV, alphasatellite, and betasatellite exhibited homogenous sequences in TW and heterogeneous sequences displaying quasispecies in PJ. Moreover, the mutation rates for PepVA were also slightly reduced in TW compared with PJ. Based on these results, we infer that the mutation of BPEV, alphasatellite, and betasatellite DNAs was strongly inhibited in TW, which is a resistant cultivar to various pepper viruses. In addition, we found that mutation rates are highly correlated with the size of the viral genome. For example, BPEV with a relatively large dsRNA genome displayed the lowest mutation rate, while DNA-A and betasatellite showed a high level of mutation rates in PJ. We provide the first evidence that the strong inhibition of the viral mutation for dsRNA and satellite DNAs in the plant cultivar leads to resistance to various viruses. We hypothesized that the quasispecies nature of satellite DNAs might be highly correlated with resistance to geminivirus infection.

In this study, we demonstrated that two different pepper cultivars were co-infected by four different viral genomes – ssRNA, dsRNA, ssDNA, and dsDNA. Furthermore, we quantified the amount of viral RNAs for an individual virus and examined the copy numbers for the respective virus. Although two transcriptomes were sequenced by different methods, there were no big differences between two cultivars for viral populations. In addition, we found that the amounts of two satellite DNAs, dsDNA, and dsRNA replications were low, indicating that their replication was very low in the pepper host.

## Conclusions

Taken together, we provide a wide range of new results associated with virus identification, viral genome assembly, viral populations, and virus quasispecies in two different plant cultivars using only pepper transcriptome data. Based on our results, it might be of interest to find a correlationship between the co-infection of different viruses and disease symptoms in the pepper host.

## Methods

### Plant materials, library preparation and sequencing

Two different pepper (*Capsicum annuum*) cultivars named Pusa Jwala (PJ) and Taiwan-2 (TW) and grown in India were used in this study. PJ and TW were identified as susceptible and resistant, respectively, to diverse viruses, including capsicum chlorosis, cucumber mosaic, and groundnut bud necrosis, which were screened by the ELISA test. Four different tissues composed of the root, stem, leaf, and fruit were pooled and used for total RNA extraction. The two prepared libraries were sequenced by the HiSeq2000 system. The PJ library was single-end sequenced, while the TW library was paired-end sequenced.

### De novo transcriptome assembly

Two raw datasets were downloaded from the SRA (Sequence Read Archive) database (http://www.ncbi.nlm.nih.gov/sra) in NCBI with their respective accession numbers. Initially, the raw data were de novo assembled by the Velvet (ver. 1.2.10) and Oases (ver. 0.2.08) programs [40] and the assembled sequences were deposited in the TSA (Transcriptome Shotgun Assembly) database with the accession numbers GAXV00000000 (PJ) and GAXU00000000 (TW). All bioinformatics analyses were conducted using a 64-core CPU and 256-GB RAM workstation installed with Xubuntu 12.04.4 LTS. The raw data were again de novo assembled by Trinity (ver. r20140717) with default parameters [41].

### Identification of viruses from transcriptome data

Four different transcriptome datasets assembled by two different de novo assemblers were subjected to a BLAST search against the virus reference database (http://www.ncbi.nlm.nih.gov/genome/viruses/). We used MEGABLAST, which is optimized for highly similar sequences with the e-value 1E-5 as a cutoff. The identified viruses were manually filtered to remove endogenous virus-like sequences and bacteriophages. Finally, virus-associated contigs from each dataset were used for further analyses.

### Assembly of consensus viral genomes

Virus-associated contigs were aligned to the respective reference viral genome using the BWA program [42] and visualized by the Tablet program [43]. In addition, we used the ClustalW program implemented in the MEGA6 program [44] followed by manual modification to obtain a consensus viral genome from each pepper cultivar. As a result, we obtained ten consensus sequences for five viral genomes from two pepper cultivars.

### Identification of a novel virus and its annotation

Our BLAST search and sequence alignment demonstrated that the 10 virus-associated contigs were derived from a single novel virus. The longest contigs were subjected to conserved domain prediction [45] and the ORF finder [46]. The complete genome sequences for PepVA were deposited in GenBank with the following accession numbers: KU923763 (PepVA isolate PJ) and KU726694 (PepVA isolate TW).

### Phylogenetic analyses

In the case of PepVA, five proteins were blasted against NCBI’s non-redundant (NR) protein database and homologous viral protein sequences were subjected to sequence alignment. The amino acid sequences for an individual viral protein were aligned by ClustalW followed by manual editing. Phylogenetic trees were constructed by 1000 bootstrap replicates using the Neighbor-Joining method with the Poisson correction of distance using the MEGA6 program [44]. For BPEV, ChiLCV DNA-A, PepLCVB, and CYVMVA, the obtained consensus nucleotide sequence from an individual cultivar were blasted against the non-redundant nucleotide (nt) database. Homologous nucleotide sequences were subjected to sequence alignment followed by manual editing. Phylogenetic trees were constructed by 1000 bootstrap replicates using the Neighbor-Joining method with the Maximum Composite Likelihood model using the MEGA6 program.

### Analysis of single nucleotide variation for each viral genome

To detect single nucleotide variations (SNVs) for the identified viral genome in each cultivar, we mapped raw sequence data on the individual consensus viral genome sequence using BWA with default parameters [42]. The aligned reads in the SAM file format were converted into BAM file format using SAMtools (ver. 1.3) [47]. The BAM file was sorted and indexed. SNV calling was performed by BCFtools (ver. 1.3), resulting in a VCF (Variant Call Format) file containing information on SNVs. The identified SNVs for each virus were visualized using the Tablet program [43].

### Viral RNAs and copy numbers

To analyze the amount of viral RNAs in each transcriptome, we performed MEGABLAST against the identified consensus viral sequences. To calculate the viral copy number for each virus, the obtained number of viral reads was multiplied by 101 and then divided by the total length of the individual genome.

## Additional files

Additional file 1: Table S1. Summary of de novo-assembled transcriptomes for two libraries using Trinity program. **Table S2.** Summary of MEGABLAST results to identify virus-associated contigs from PJ transcriptome. **Table S3.** Summary of MEGABLAST results to identify virus-associated contigs from TW transcriptome. **Table S4.** Summary of identified viruses in PJ and TW transcriptomes with numbers of associated viral contigs. **Table S5.** Single nucleotide variations of BPEV in the PJ transcriptome. **Table S6.** Single nucleotide variations of BPEV in the TW transcriptome. **Table S7.** Single nucleotide variations of PepVA in the PJ transcriptome. **Table S8.** Single nucleotide variations of PepVA in the TW transcriptome. **Table S9.** Single nucleotide variations of ChiLCV DNA-A in the PJ transcriptome. **Table S10.** Single nucleotide variations of ChiLCV DNA-A in the TW transcriptome. **Table S11.** Single nucleotide variations of PepLCV betasatellite in the PJ transcriptome. **Table S12.** Single nucleotide variations of PepLCV betasatellite in the TW transcriptome. **Table S13.** Single nucleotide variations of CYVMV alphasatellite in the PJ transcriptome. **Table S14.** Single nucleotide variations of CYVMV alphasatellite in the TW transcriptome. **Table S15.** Number of viral sequence reads and copy number for identified viruses. (XLSX 131 kb)

Additional file 2: Figure S1. Alignment of contigs associated with TVCV. Green and orange colored lines and bars indicate contigs assembled by Velvet and Trinity, respectively. The genome organizations of TVCV were manually drawn based on the reference viral genome annotation. Putative ORFs were indicated. Abbreviations: movement protein (MP) and transactivator factor (TF). **Figure S2** Sequence alignment for PepLCVB**.** C1 nucleotide and protein sequences were obtained from PJ and TW transcriptomes and used for sequence alignment with C1 reference sequence. Alignment was conducted and visualized by ClustalW. **Figure S3** Sequence alignment for CYVMVA. Replication gene and protein sequences for CYVMVA were obtained from PJ and TW transcriptomes and used for sequence alignment with the alphasatellite replication reference sequence. Alignment was conducted and visualized by ClustalW. **Figure S4** Phylogenetic relationships of ORF2, ORF3, and ORF5 with respective other homologous viral proteins for PepVA. (PDF 1867 kb)

## Abbreviations

AcLV: Aconitum latent virus
ALPV: Aphid lethal paralysis virus
BPEV: Bell pepper endornavirus
ChiLCV: Chilli leaf curl virus
CYVMVA: Croton yellow vein mosaic virus alphasatellite
ds: Double-stranded
GaILV: Gaillardia latent virus
NGS: Next generation sequencing
PepLCBV: Pepper leaf curl Bangladesh virus
PepLCVB: Pepper leaf curl virus betasatellite
PeSV: Pea streak virus
PJ: Pusa Jwala
PVP: Potato virus P
SNVs: Single nucleotide variations
ss: Single-stranded
TGB: Triple-gene-block
TVCV: Tobacco vein clearing virus
TW: Taiwan2
TYLCV: Tomato yellow leaf curl virus;

## References

[1] Aguilar-Meléndez A, Morrell PL, Roose ML, Kim S-C (2009). Genetic diversity and structure in semiwild and domesticated chiles (*Capsicum annuum*; Solanaceae) from Mexico. Am J Bot.

[2] Kenyon L, Kumar S, Tsai W-S, JdA H (2014). Virus Diseases of Peppers (Capsicum spp.) and Their Control. Control of Plant Virus Diseases: Seed-Propagated Crops.

[3] Mansoor S, Briddon RW, Zafar Y, Stanley J (2003). Geminivirus disease complexes: an emerging threat. Trends Plant Sci.

[4] Barba M, Czosnek H, Hadidi A (2014). Historical perspective, development and applications of next-generation sequencing in plant virology. Viruses.

[5] Alabi O, Al Rwahnih M, Jifon J, Gregg L, Crosby K, Mirkov T. First Report of Pepper vein yellows virus Infecting Pepper (Capsicum spp.) in the United States. Plant Dis. 2015;99(11):1656. https://doi.org/10.1094/PDIS-03-15-0329-PDN.

[6] Jo Y, Choi H, Cho WK (2015). De novo assembly of a bell pepper endornavirus genome sequence using RNA sequencing data. Genome announcements.

[7] Chen B, Bernards M, Wang A (2015). Complete genome sequence of a bell pepper endornavirus isolate from Canada. Genome announcements.

[8] Dombrovsky A, Glanz E, Lachman O, Sela N, Doron-Faigenboim A, Antignus Y (2013). The complete genomic sequence of Pepper yellow leaf curl virus (PYLCV) and its implications for our understanding of evolution dynamics in the genus Polerovirus. PLoS One.

[9] Lauring AS, Andino R (2010). Quasispecies theory and the behavior of RNA viruses. PLoS Pathog.

[10] Domingo E, Sheldon J, Perales C (2012). Viral quasispecies evolution. Microbiol Mol Biol Rev.

[11] García-Arenal F, Fraile A, Malpica JM (2001). Variability and genetic structure of plant virus populations. Annu Rev Phytopathol.

[12] Schneider WL, Roossinck MJ (2001). Genetic diversity in RNA virus quasispecies is controlled by host-virus interactions. J Virol.

[13] Duffy S, Holmes EC (2008). Phylogenetic evidence for rapid rates of molecular evolution in the single-stranded DNA begomovirus tomato yellow leaf curl virus. J Virol.

[14] Jo Y, Choi H, Cho JK, Yoon J-Y, Choi S-K, Cho WK. In silico approach to reveal viral populations in grapevine cultivar Tannat using transcriptome data. Sci Rep. 2015;5:15841. https://doi.org/10.1038/srep15841. 10.1038/srep15841 PMC4623741 26508692

[15] Coetzee B, Freeborough M-J, Maree HJ, Celton J-M, Rees DJG, Burger JT (2010). Deep sequencing analysis of viruses infecting grapevines: virome of a vineyard. Virology.

[16] Wylie SJ, Luo H, Li H, Jones MG (2012). Multiple polyadenylated RNA viruses detected in pooled cultivated and wild plant samples. Arch Virol.

[17] Wylie SJ, Li H, Saqib M, Jones MG (2014). The global trade in fresh produce and the vagility of plant viruses: A case study in garlic. PLoS One.

[18] Gu Y-H, Tao X, Lai X-J, Wang H-Y, Zhang Y-Z (2014). Exploring the polyadenylated RNA virome of sweet potato through high-throughput sequencing. PloS one.

[19] Jo Y, Choi H, Yoon J-Y, Choi S-K, Cho WK (2016). In silico identification of Bell pepper endornavirus from pepper transcriptomes and their phylogenetic and recombination analyses. Gene.

[20] Kumar S, Kumar R, Kumar S, Kumar Singh A, Singh M, Bahadur Rai A, et al. Incidence of leaf curl disease on Capsicum germplasm under field conditions. Indian Journal of Agricultural Sciences. 2011;81:187.

[21] Kumar S, Kumar S, Singh M, Singh AK, Rai M (2006). Identification of host plant resistance to pepper leaf curl virus in chilli (Capsicum species). Sci Hortic.

[22] Hagen C, Frizzi A, Gabriels S, Huang M, Salati R, Gabor B, et al. Accurate and sensitive diagnosis of geminiviruses through enrichment, high-throughput sequencing and automated sequence identification. Arch Virol. 2012;157:907–15. 10.1007/s00705-012-1253-7 22327393

[23] Idris A, Al-Saleh M, Piatek MJ, Al-Shahwan I, Ali S, Brown JK (2014). Viral metagenomics: Analysis of begomoviruses by illumina high-throughput sequencing. Viruses.

[24] Seguin J, Rajeswaran R, Malpica-Lopez N, Martin RR, Kasschau K, Dolja VV, et al. De novo reconstruction of consensus master genomes of plant RNA and DNA viruses from siRNAs. PLoS One. 2014;9:e88513. 10.1371/journal.pone.0088513 PMC3921208 24523907

[25] Jo Y, Choi H, Cho WK (2015). Complete genome sequence of a hop latent virus infecting hop plants. Genome announcements.

[26] Isnard M, Granier M, Frutos R, Reynaud B, Peterschmitt M (1998). Quasispecies nature of three maize streak virus isolates obtained through different modes of selection from a population used to assess response to infection of maize cultivars. J Gen Virol.

[27] Ge L, Zhang J, Zhou X, Li H (2007). Genetic structure and population variability of tomato yellow leaf curl China virus. J Virol.

[28] Kutnjak D, Rupar M, Gutierrez-Aguirre I, Curk T, Kreuze JF, Ravnikar M (2015). Deep sequencing of virus-derived small interfering RNAs and RNA from viral particles shows highly similar mutational landscapes of a plant virus population. J Virol.

[29] Glouzon J-PS, Bolduc F, Wang S, Najmanovich RJ, Perreault J-P (2014). Deep-sequencing of the peach latent mosaic viroid reveals new aspects of population heterogeneity. PLoS One.

[30] Van den Hoecke S, Verhelst J, Vuylsteke M, Saelens X (2015). Analysis of the genetic diversity of influenza A viruses using next-generation DNA sequencing. BMC Genomics.

[31] Jo Y, Choi H, Cho WK. Genome Sequence of Dengue virus 3 from the Pythium insidiosum Transcriptomes. Front Microbiol. 2016;7:926. https://doi.org/10.3389/fmicb.2016.00926. 10.3389/fmicb.2016.00926 PMC4908670 27379056

[32] Ng JC, Perry KL (2004). Transmission of plant viruses by aphid vectors. Mol Plant Pathol.

[33] Bedford ID, Briddon RW, Brown JK, Rosell R, Markham PG (1994). Geminivirus transmission and biological characterisation of *Bemisia tabaci* (Gennadius) biotypes from different geographic regions. Ann Appl Biol.

[34] Okada R, Kiyota E, Sabanadzovic S, Moriyama H, Fukuhara T, Saha P, et al. Bell pepper endornavirus: molecular and biological properties, and occurrence in the genus Capsicum. J Gen Virol. 2011;92:2664–73. 10.1099/vir.0.034686-0 21775578

[35] Lockhart B, Menke J, Dahal G, Olszewski N (2000). Characterization and genomic analysis of tobacco vein clearing virus, a plant pararetrovirus that is transmitted vertically and related to sequences integrated in the host genome. J Gen Virol.

[36] Kil E-J, Kim S, Lee Y-J, Byun H-S, Park J, Seo H, et al. Tomato yellow leaf curl virus (TYLCV-IL): a seed-transmissible geminivirus in tomatoes. Scientific reports. 2016;6 10.1038/srep19013 PMC4705557 26743765

[37] Rentería-Canett I, Xoconostle-Cázares B, Ruiz-Medrano R, Rivera-Bustamante RF (2011). Geminivirus mixed infection on pepper plants: synergistic interaction between PHYVV and PepGMV. Virol J.

[38] Roossinck MJ (2011). The good viruses: viral mutualistic symbioses. Nat Rev Microbiol.

[39] Roossinck MJ (2015). A new look at plant viruses and their potential beneficial roles in crops. Mol Plant Pathol.

[40] Schulz MH, Zerbino DR, Vingron M, Birney E (2012). Oases: robust de novo RNA-seq assembly across the dynamic range of expression levels. Bioinformatics.

[41] Grabherr MG, Haas BJ, Yassour M, Levin JZ, Thompson DA, Amit I, et al. Full-length transcriptome assembly from RNA-Seq data without a reference genome. Nat Biotechnol. 2011;29:644–52. 10.1038/nbt.1883 PMC3571712 21572440

[42] Li H, Durbin R (2009). Fast and accurate short read alignment with Burrows–Wheeler transform. Bioinformatics.

[43] Milne I, Stephen G, Bayer M, Cock PJ, Pritchard L, Cardle L, et al. Using Tablet for visual exploration of second-generation sequencing data. Briefings Bioinf. 2013;14(2):193–202. https://doi.org/10.1093/bib/bbs012. 10.1093/bib/bbs012 22445902

[44] Tamura K, Stecher G, Peterson D, Filipski A, Kumar S. MEGA6: molecular evolutionary genetics analysis version 6.0. Mol Biol Evol. 2013;30(12):2725–29. https://doi.org/10.1093/molbev/mst197. 10.1093/molbev/mst197 PMC3840312 24132122

[45] Marchler-Bauer A, Lu S, Anderson JB, Chitsaz F, Derbyshire MK, DeWeese-Scott C, et al. CDD: a Conserved Domain Database for the functional annotation of proteins. Nucleic Acids Res. 2011;39:D225–9. 10.1093/nar/gkq1189 PMC3013737 21109532

[46] Rombel IT, Sykes KF, Rayner S, Johnston SA (2002). ORF-FINDER: a vector for high-throughput gene identification. Gene.

[47] Li H, Handsaker B, Wysoker A, Fennell T, Ruan J, Homer N, et al. The sequence alignment/map format and SAMtools. Bioinformatics. 2009;25:2078–9. 10.1093/bioinformatics/btp352 PMC2723002 19505943

