# Supplementary material for: The pepper virome: natural co-infection of diverse viruses and their quasispecies
Source: BMC Genomics. 2017 Jun 8;18:453. doi: 10.1186/s12864-017-3838-8 (PMC5465472; doi:10.1186/s12864-017-3838-8)
Supplement: Supplementary file 2 — Alignment of contigs associated with TVCV. Green and orange colored lines and bars indicate contigs assembled by Velvet and Trinity, respectively. The genome organizations of TVCV were manually drawn based on the reference viral genome annotation. Putative ORFs were indicated. Abbreviations: movement protein (MP) and transactivator factor (TF). Figure S2 Sequence alignment for PepLCVB. C1 nucleotide and protein sequences were obtained from PJ and TW transcriptomes and used for sequence alignment with C1 reference sequence. Alignment was conducted and visualized by ClustalW. Figure S3 Sequence alignment for CYVMVA. Replication gene and protein sequences for CYVMVA were obtained from PJ and TW transcriptomes and used for sequence alignment with the alphasatellite replication reference sequence. Alignment was conducted and visualized by ClustalW. Figure S4 Phylogenetic relationships of ORF2, ORF3, and ORF5 with respective other homologous viral proteins for PepVA. (PDF 1867 kb) [file 12864_2017_3838_MOESM2_ESM.pdf]

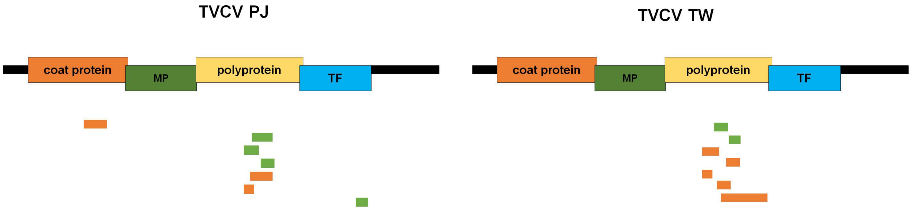
TVCV PJ
coat protein
MP
polyprotein
TF
TVCV TW
coat protein
MP
polyprotein
TF

PepLCVB C1 gene PJ

```
C1              ATGCAAATGACGATCAAGTATACTAACGAGAAGGGTATGGTATTTACCATCAACGTAAAG    60
GAXV01013424.1  ATGCAAATGACGATCAAGTATACTAATGAGAAGGGTATGCTATTTACCATCAACGTGAAG    60
GAXV01013423.1  ATGCAAATGACGATCAAGTATACTAATGAGAAGGGTATGCTATTTACCATCAACGTGAAG    60
c19466_g1_i1    ATGCAAATGACGATCAAGTATACTAACGAGAAGGGGATGGTATTTACCATCGACGTGAAG    60
                ************************** ******** *** *********** **** ***

C1              ATCAACGAGGACAATTCCATTCTGGTCCGGATTGACTTGTGTTCGACAAAATCACCAGCC    120
GAXV01013424.1  ATCCACGACGACAATTCACTTCTTGTCCGGATTGATTTGTGCTCAACAAGATCACCAGCC    120
GAXV01013423.1  ATCCACGACGACAATTCACTTCTTGTCCGGATTGATTTGTGCTCAACAAGATCACCAGCC    120
c19466_g1_i1    ATAAACGATGACAGTTCAATTCTTGTCCGGATTGAATTGTGCTCGACAAGATCACCAGCC    120
                **  **** **** ***  **** *********** ***** ** **** **********

C1              CTGGCAAAGAAGACCTTCATGATCCCATACGGACATGATGGGATCATACAACCATTCGAC    180
GAXV01013424.1  CTGACGAAGAAGACCTTCATGATCCCATACGGCCATGATGGGATCATACCACCGTTCGAC    180
GAXV01013423.1  CTGACGAAGAAGACCTTCATGATCCCATACGGCCATGATGGGATCATACCACCGTTCGAC    180
c19466_g1_i1    CTGGCTAAGAAGACCTTCATGATCCCATACGGCCATGATGGGATCATACCACCTTTCGAC    180
                *** * ************************** **************** *** ******

C1              TTCAACAATTTGGAGGAAGGGATACATCATATATTGAAGGTCATGTACAAAGATTCTCGT    240
GAXV01013424.1  TTCAACAATTTAGAGGAAGGACTACATAATATATTGAAGGTCATGTACAGGGATTGTCAT    240
GAXV01013423.1  TTCAACAATTTAGAGGAAGGACTACATAATATATTGAAGGTCATGTACAGGGATTGTCAT    240
c19466_g1_i1    TTCAACAATTTGGAGGACGGGATACATAATATATTAAAGGTCATGTACAGGGATTGCCAT    240
                *********** ***** **  ***** ******* *************  ****  * *

C1              ATTTGGGAGTTTCGACAGGAGGACATGGTAGAGGCTATTGATATAATGATGATGCATGAA    300
GAXV01013424.1  GTTAGGGAGTTTCGACAGGAGGACATGGTAGAGGCTATTGATATCATGATGCTGCATGAA    300
GAXV01013423.1  GTTAGGGAGTTTCGACAGGAGGACATGGTAGAGGCTATTGATATCATGATGCTGCATGAA    300
c19466_g1_i1    GTTGGGGAGTTTCGACAGGAGGATATGGTAGAGTCTATTGATATATTGATGATGCATGAG    300
                 ** ******************* ********* **********  ***** ******* 

C1              GCACCATTAGTTGATATACGTATAGGGGATGAATATGATGTCTGTACGAATGTGTGTGTG    360
GAXV01013424.1  GCACCAGTAATTGATATACGTATAGCGGATGAATATGATGTATGTACGACTGTGTGTGTG    360
GAXV01013423.1  GCACCAGTAATTGATATACGTATAGCGGATGAATATGATGTATGTACGACTGTGTGTGTG    360
c19466_g1_i1    GCCCCATTAGTTGATATACGTATAGCGGATGAATATGATGTATGTACGAATGTGTGTGTG    360
                ** *** ** *************** *************** ******* **********

C1              TGA       363
GAXV01013424.1  TGA       363
GAXV01013423.1  TGA       363
c19466_g1_i1    TGA       363
                ***
```

PepLCVB C1 gene TW

```
c47706_g1_i1    ATGCAAATGACGATCAAGTATACTAATGAGAAGGGTATGCTATTTACCATCAACGTGAAG  60
C1              ATGCAAATGACGATCAAGTATACTAACGAGAAGGGTATGGTATTTACCATCAACGTAAAG  60
c44565_g2_i2    ATGCAAATGACGATCAAGTATACTAACGAGAAGGGGATGGTATTTACCATCGACGTGAAG  60
c47706_g1_i3    ATGCAAATGACGATCAAGTATACTAACGAGAAGGGGATGGTATTTACCATCGACGTGAAG  60
                ************************** ******** *** *********** **** ***

c47706_g1_i1    ATCCACGACGACAATTCACTTCTTGTCCGGATTGATTTGTGCTCAACAAGATCACCAGCC  120
C1              ATCAACGAGGACAATTCCATTCTGGTCCGGATTGACTTGTGTTCGACAAAATCACCAGCC  120
c44565_g2_i2    ATAAACGATGACAGTTCAATTCTTGTCCGGATTGAATTGTGCTCGACAAGATCACCAGCC  120
c47706_g1_i3    ATAAACGATGACAGTTCAATTCTTGTCCGGATTGAATTGTGCTCGACAAGATCACCAGCC  120
                **  **** **** ***  **** *********** ***** ** **** **********

c47706_g1_i1    CTGACGAAGAAGACCTTCATGATCCCATACGGCCATGATGGGATCATACCACCGTTCGAC  180
C1              CTGGCAAAGAAGACCTTCATGATCCCATACGGACATGATGGGATCATACAACCATTCGAC  180
c44565_g2_i2    CTGGCTAAGAAGACCTTCATGATCCCATACGGCCATGATGGGATCATACCACCTTTCGAC  180
c47706_g1_i3    CTGGCTAAGAAGACCTTCATGATCCCATACGGCCATGATGGGATCATACCACCTTTCGAC  180
                *** * ************************** **************** *** ******

c47706_g1_i1    TTCAACAATTTAGAGGAAGGACTACATAATATATTGAAGGTCATGTACAGGGATTGTCAT  240
C1              TTCAACAATTTGGAGGAAGGGATACATCATATATTGAAGGTCATGTACAAAGATTCTCGT  240
c44565_g2_i2    TTCAACAATTTGGAGGACGGGATACATAATATATTAAAGGTCATGTACAGGGATTGCCAT  240
c47706_g1_i3    TTCAACAATTTGGAGGACGGGATACATAATATATTAAAGGTCATGTACAGGGATTGCCAT  240
                *********** ***** **  ***** ******* *************  ****  * *

c47706_g1_i1    GTTAGGGAGTTTCGCCAGGAGGACATGGTAGAGGCTATTGATATCATGATGCTGCATGAA  300
C1              ATTTGGGAGTTTCGACAGGAGGACATGGTAGAGGCTATTGATATAATGATGATGCATGAA  300
c44565_g2_i2    GTTGGGGAGTTTCGACAGGAGGATATGGTAGAGTCTATTGATATATTGATGATGCATGAG  300
c47706_g1_i3    GTTGGGGAGTTTCGCCAGGAGGATATGGTAGAGTCTATTGATATATTGATGATGCATGAG  300
                 ** ********** ******** ********* **********  ***** ******* 

c47706_g1_i1    GCACCAGTAATTGATATACGTATAGCGGATGAATATGATGTATGTACGACTGTGTGTGTG  360
C1              GCACCATTAGTTGATATACGTATAGGGGATGAATATGATGTCTGTACGAATGTGTGTGTG  360
c44565_g2_i2    GCCCCATTAGTTGATATACGTATAGGGGATGAATATGATGTATGTACAAATGTGTGTGTC  360
c47706_g1_i3    GCCCCATTAGTTGATATACGTATAGGGGATGAATATGATGTATGTACAAATGTGTGTGTC  360
                ** *** ** *************** *************** ***** * ********* 

c47706_g1_i1    TGA       363
C1              TGA       363
c44565_g2_i2    TGA       363
c47706_g1_i3    TGA       363
                ***
```

PepLCVB C1 protein PJ

```
GAXV01013424.1  MQMTIKYTNEKGMLFTINVKIHDDNSLLVRIDLCSTRSPALTKKTFMIPYGHDGIIPPFD  60
GAXV01013423.1  MQMTIKYTNEKGMLFTINVKIHDDNSLLVRIDLCSTRSPALTKKTFMIPYGHDGIIPPFD  60
C1              MQMTIKYTNEKGMVFTINVKINEDNSILVRIDLCSTKSPALAKKTFMIPYGHDGIIQPFD  60
c19466_g1_i1    MQMTIKYTNEKGMVFTIDVKINDDSSILVRIELCSTRSPALAKKTFMIPYGHDGIIPPFD  60
                *************:***:***.:*.*:****:****:****:************** ***

GAXV01013424.1  FNNLEEGLHNILKVMYRDCHVREFRQEDMVEAIDIMMLHEAPVIDIRIADEYDVCTTVCV  120
GAXV01013423.1  FNNLEEGLHNILKVMYRDCHVREFRQEDMVEAIDIMMLHEAPVIDIRIADEYDVCTTVCV  120
C1              FNNLEEGIHHILKVMYKDSRIWEFRQEDMVEAIDIMMMHEAPLVDIRIGDEYDVCTNVCV  120
c19466_g1_i1    FNNLEDGIHNILKVMYRDCHVGEFRQEDMVESIDILMMHEAPLVDIRIADEYDVCTNVCV  120
                *****:*:*.******:*.:: *********:***:*:****::****.*******.***

GAXV01013424.1  *          120
GAXV01013423.1  *          120
C1              *          120
c19466_g1_i1    *          120
                *
```

PepLCVB C1 protein TW

```
c47706_g1_i1    MQMTIKYTNEKGMLFTINVKIHDDNSLLVRIDLCSTRSPALTKKTFMIPYGHDGIIPPFD   60
C1              MQMTIKYTNEKGMVFTINVKINEDNSILVRIDLCSTKSPALAKKTFMIPYGHDGIIQPFD   60
c44565_g2_i2    MQMTIKYTNEKGMVFTIDVKINDDSSILVRIELCSTRSPALAKKTFMIPYGHDGIIPPFD   60
c47706_g1_i3    MQMTIKYTNEKGMVFTIDVKINDDSSILVRIELCSTRSPALAKKTFMIPYGHDGIIPPFD   60
                *************:***:***.:*.*:****:****:****:************** ***

c47706_g1_i1    FNNLEEGLHNILKVMYRDCHVREFRQEDMVEAIDIMMLHEAPVIDIRIADEYDVCTTVCV   120
C1              FNNLEEGIHHILKVMYKDSRIWEFRQEDMVEAIDIMMMHEAPLVDIRIGDEYDVCTNVCV   120
c44565_g2_i2    FNNLEDGIHNILKVMYRDCHVGEFRQEDMVESIDILMMHEAPLVDIRIGDEYDVCTNVCV   120
c47706_g1_i3    FNNLEDGIHNILKVMYRDCHVGEFRQEDMVESIDILMMHEAPLVDIRIGDEYDVCTNVCV   120
                *****:*:*.******:*.:: *********:***:*:****::****.*******.***

c47706_g1_i1    * 120
C1              * 120
c44565_g2_i2    * 120
c47706_g1_i3    * 120
                *
```

CYVMVA replication gene PJ

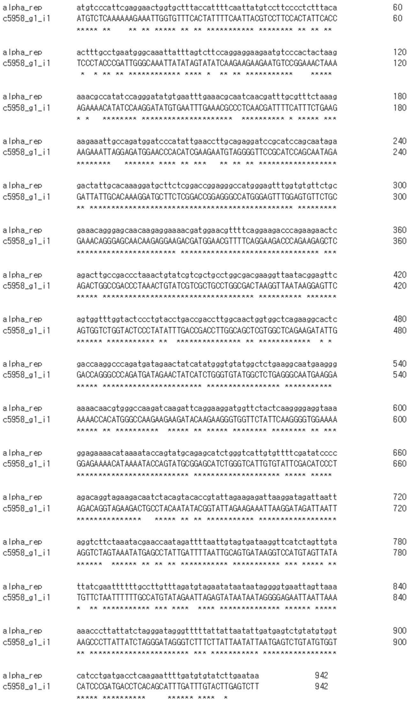

CYVMVA replication gene TW

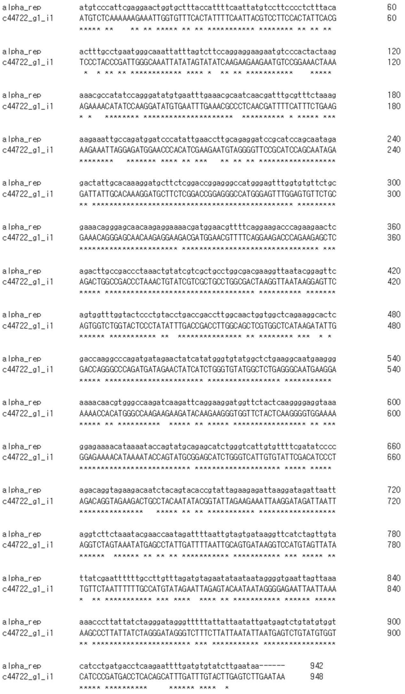

CYVMVA replication protein PJ

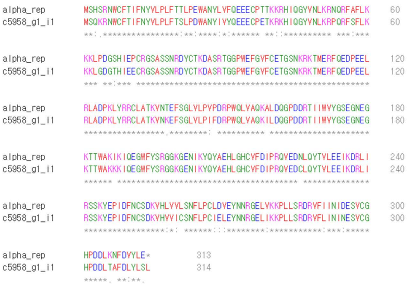

CYVMVA replication protein TW

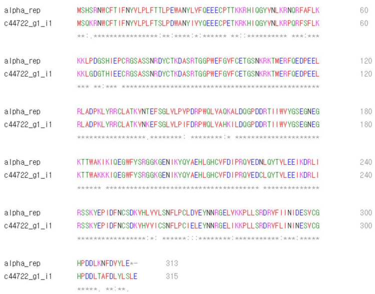

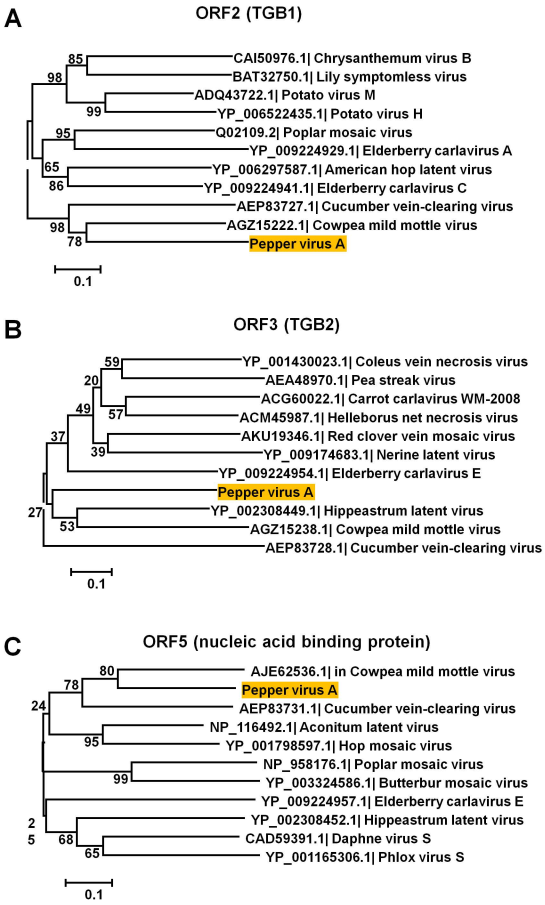
A
ORF2 (TGB1)
85
98
99
95
65
86
98
78
CAI50976.1| Chrysanthemum virus B
BAT32750.1| Lily symptomless virus
ADQ43722.1| Potato virus M
YP_006522435.1| Potato virus H
Q02109.2| Poplar mosaic virus
YP_009224929.1| Elderberry carlavirus A
YP_006297587.1| American hop latent virus
YP_009224941.1| Elderberry carlavirus C
AEP83727.1| Cucumber vein-clearing virus
AGZ15222.1| Cowpea mild mottle virus
Pepper virus A
0.1
B
ORF3 (TGB2)
59
20
49
57
37
39
27
53
YP_001430023.1| Coleus vein necrosis virus
AEA48970.1| Pea streak virus
ACG60022.1| Carrot carlavirus WM-2008
ACM45987.1| Helleborus net necrosis virus
AKU19346.1| Red clover vein mosaic virus
YP_009174683.1| Nerine latent virus
YP_009224954.1| Elderberry carlavirus E
Pepper virus A
YP_002308449.1| Hippeastrum latent virus
AGZ15238.1| Cowpea mild mottle virus
AEP83728.1| Cucumber vein-clearing virus
0.1
C
ORF5 (nucleic acid binding protein)
80
78
24
95
99
2
5
68
65
AJE62536.1| in Cowpea mild mottle virus
Pepper virus A
AEP83731.1| Cucumber vein-clearing virus
NP_116492.1| Aconitum latent virus
YP_001798597.1| Hop mosaic virus
NP_958176.1| Poplar mosaic virus
YP_003324586.1| Butterbur mosaic virus
YP_009224957.1| Elderberry carlavirus E
YP_002308452.1| Hippeastrum latent virus
CAD59391.1| Daphne virus S
YP_001165306.1| Phlox virus S
0.1
